# A bifunctional peptide–selenium nanocomposite for lysosomal degradation of PD-L1 and enhanced cancer immunotherapy

**DOI:** 10.3389/fimmu.2025.1678911

**Published:** 2025-10-29

**Authors:** Yang Wang, Jun Feng, Jin Yan, Weiming You, Siqi Yan

**Affiliations:** ^1^ Department of Hepatology, The Second Affiliated Hospital of Xi’an Jiaotong University, Xi’an, Shaanxi, China; ^2^ Department of Tumor and Immunology in Precision Medical Institute, Western China Science and Technology Innovation Port, The Second Affiliated Hospital of Xi’an Jiaotong University, Xi’an, Shaanxi, China; ^3^ Institute for Stem Cell and Regenerative Medicine, The Second Affiliated Hospital of Xi’an Jiaotong University, Xi’an, Shaanxi, China

**Keywords:** PD-L1, peptide, selenium nanoparticles, lysosomal degradation, immunotherapy, tumor immune microenvironment

## Abstract

**Background:**

Immune checkpoint blockade (ICB) therapies that inhibit PD-1/PD-L1 signaling have revolutionized oncology, yet their benefits are constrained by limited penetration into tumor tissues, inability to eliminate intracellular PD-L1, and the emergence of resistance pathways. Approaches aimed at promoting intracellular PD-L1 degradation and reshaping the tumor immune microenvironment hold promise for overcoming these therapeutic barriers.

**Methods:**

A bifunctional therapeutic peptide a capable of binding cytosolic PD-L1 and the molecular chaperone HSC70 was synthesized to facilitate chaperone-mediated autophagy–dependent lysosomal degradation of PD-L1. To improve stability and tumor delivery, peptide a was self-assembled with nano-selenium to form SA. SA was characterized by TEM, DLS, and UV–vis spectroscopy. Binding affinity was validated by ITC. Cellular uptake, PD-L1 degradation, and lysosomal trafficking were assessed via flow cytometry, western blotting, and immunofluorescence. Antitumor efficacy was evaluated in CT26 models and MC38 spheroid assays, with mechanistic analysis performed using immunohistochemistry and flow cytometry. Safety was comprehensively assessed.

**Results:**

SA exhibited uniform spherical morphology (~35 nm) and excellent stability. *In vitro* studies demonstrated enhanced cellular uptake compared to free peptide and dose-dependent PD-L1 degradation (31.1% reduction at 0.6 μg/mL), which was significantly attenuated by lysosomal inhibition, confirming the chaperone-mediated autophagy (CMA)-dependent mechanism. Immunofluorescence analysis revealed enhanced colocalization of PD-L1 with HSC70 and LAMP2-positive lysosomes following SA treatment. *In vivo*, SA achieved 88.72% tumor growth inhibition, surpassing anti-PD-L1 antibody treatment (66.97%). SA also demonstrated superior efficacy in MC38 tumor spheroid assays across multiple time points (48h and 72h). Mechanistically, SA downregulated PD-L1, increased CD8^+^ T cell infiltration 9.4-fold, reduced regulatory T cells by 47.81%, and enhanced cytotoxic CD8^+^ T cell function with Granzyme B and IFN-γ populations increased 6.8-fold and 2.9-fold, respectively. Comprehensive safety evaluation revealed no treatment-related toxicity, with stable body weight, normal hematological parameters, preserved organ histology, and balanced serum cytokine profiles throughout the study period.

**Conclusions:**

SA represents a novel intracellular PD-L1–targeted nanoplatform that promotes lysosome-mediated PD-L1 clearance, remodels the tumor immune milieu, and demonstrates superior antitumor performance compared to PD-L1 antibodies. This dual mechanism addresses key limitations of current ICB therapies and supports further clinical translation.

## 1 Introduction

Cancer immunotherapy, particularly the use of monoclonal antibodies that target immune checkpoint pathways such as PD-1 and PD-L1, has significantly transformed the treatment landscape for various malignancies (1–6). Despite notable clinical achievements, these treatments face several inherent limitations, including poor tumor tissue penetration (7), inability to target intracellular immune checkpoints (8), high immunogenicity (9), severe immune-related adverse events (irAEs) (10) and the necessity for intravenous administration (11). These constraints—particularly inadequate tumor microenvironment (TME) infiltration—significantly restrict their clinical efficacy and broad applicability, especially in solid tumors.

PD-L1, a critical immune checkpoint protein frequently overexpressed on malignant cells, promotes immune escape by interacting with PD-1 on T lymphocytes and dampening T cell–mediated antitumor responses (12). Although commercially available PD-L1 antibodies can efficiently block extracellular PD-1/PD-L1 interactions, they generally fail to eliminate intracellular reservoirs of PD-L1. Importantly, internalized PD-L1 can recycle to the cell surface, sustaining immunosuppressive signaling and contributing to resistance against immune checkpoint blockade therapies (13). Accordingly, strategies aimed at promoting the comprehensive intracellular degradation of PD-L1 have garnered increasing interest as a means to overcome this resistance (14).

Among intracellular degradation mechanisms, lysosomal pathways—particularly chaperone-mediated autophagy (CMA) has attracted significant attention for their role in regulating PD-L1 turnover. For instance, the molecular chaperone HSC70 has been shown to drive PD-L1 breakdown through the endosome–lysosome trafficking pathway while preventing its return to the plasma membrane (15). This process relies on the direct engagement of HSC70 with PD-L1, which disrupts the stabilizing association between PD-L1 and its regulatory partner CMTM6. In parallel, research led by Xu Jie demonstrated that the adaptor protein HIP1R can directly bind with PD-L1 and direct it toward lysosomal clearance through a lysosome-targeting motif (16). Despite the therapeutic promise of enhancing HSC70-or HIP1R-mediated PD-L1 degradation, two key factors mechanisms limit their effectiveness. First, HIP1R is often downregulated in tumor, leading to PD-L1 accumulation, weakened cytotoxic T cell responses, and enhanced immune escape (16, 17). Second, the membrane-associated oncoprotein CMTM6, a member of the CMTM family, binds PD-L1 and shields it from lysosomal breakdown. CMTM6 interacts with the regulatory domain of PD-L1 and competitively obstructs HSC70 binding, thereby stabilizing PD-L1 at the cell surface (18). These observations emphasize the critical demand for alternative strategies that can circumvent these resistance pathways and enhance the effectiveness of PD-L1–targeted immunotherapy.

To tackle these obstacles, we constructed a dual-targeting peptide (referred to as peptide a), designed to simultaneously engage HSC70 and cytosolic PD-L1—the principal source of membrane-localized PD-L1. By selectively targeting cytoplasmic PD-L1, peptide a bypasses CMTM6-mediated competitive inhibition, thereby overcoming a major resistance mechanism and decreasing the total PD-L1 pool within cancers. To further improve the therapeutic potency and intracellular delivery of such peptides, nanoparticle-based delivery systems have been increasingly employed (19–21). Among these, nano-selenium carriers exhibit several advantageous properties, including excellent biocompatibility, intrinsic immunomodulatory and antioxidative functions, and high delivery efficiency (22–24). Here, we engineered a nano-selenium–based platform to deliver PD-L1-degrading peptides directly to tumor cells. This system is specifically designed to enhance intracellular peptide stability, facilitate deeptumor penetration, and augment anti-tumor immune responses by promoting lysosomal and CMA-dependent PD-L1 degradation. Besides, to rigorously validate this intracellular targeting strategy in a clinically relevant yet therapeutically challenging context, we deliberately selected CT26, a proficient mismatch repair (pMMR)/microsatellite stable (MSS) colorectal carcinoma that is intrinsically resistant to PD-1/PD-L1 antibodies (clinical response rate <5%) (25, 26). Unlike MSI-H tumors, pMMR/MSS tumors—comprising ~85% of colorectal cancers—exhibit sustained PD-L1 recycling that maintains immunosuppression despite surface blockade, providing a stringent model to test whether comprehensive intracellular degradation can overcome the resistance mechanisms where conventional antibodies fail (15, 17, 27). Through this nano-selenium–peptide platform, we demonstrate effective intracellular checkpoint modulation that achieves superior therapeutic efficacy compared to conventional PD-L1 monoclonal antibodies, offering a promising and innovative approach to advance the next generation of cancer immunotherapies, particularly for checkpoint inhibitor-refractory tumors.

## 2 Materials and methods

### 2.1 Synthesis of SA

Peptide a was meticulously synthesized on 2-Chlorotrityl chloride resin, utilizing an optimized Fmoc-based HBTU activation protocol coupled with *in situ* neutralization via DIEA, executed on automated peptide synthesizer (CS Bio 336X) with HCTU/HOBt coupling. Following cleavage and deprotection—accomplished through a reagent mixture containing 88% trifluoroacetic acid (TFA), alongside 5% phenol, 5% deionized water, and 2% triisopropylsilane (TIPS)—the resultant crude peptide underwent precipitation using cold diethyl ether before being purified by semi-preparative C18 column, and its molecular weight of the purified compound was subsequently confirmed through electrospray ionization mass spectrometry (ESI-MS).

For SA preparation, 40 μL of 0.1 M selenous acid solution was combined with 0.4 mg of polyvinylpyrrolidone (PVP, possessing an average molecular weight of 10,000), and 2 mg of peptide a in 7.96 mL of deionized water. Following this initial blending, the mixture was enhanced by the addition of 2 mL freshly prepared ascorbic acid aqueous solution (0.1 M), initiating a reduction reaction that was judiciously conducted at a stirring rate of 500 rpm for a duration of 30 minutes using a magnetic stirrer. Subsequently, to purify the reaction mixture and eliminate any unreacted components, it underwent dialysis against distilled water employing a dialysis membrane with an appropriate molecular weight cutoff set at 3,500 Da. This process ultimately yielded the targeted SA solution in its refined form.

### 2.2 Characterization of SA

The morphology and crystalline lattice of the nanoparticles were examined by transmission electron microscopy (TEM) on a Talos L120C G2 instrument operated at 120 kV. The hydrodynamic size distribution was determined through dynamic light scattering (DLS) using a Malvern Zetasizer Nano ZS system. To evaluate colloidal stability, the formulations were incubated in PBS buffer enriched with 10% serum at 37°C, and particle size distributions were measured across various time points to comprehensively investigate nanoparticle aggregation phenomena. The surface chemical architecture of the peptide-modified selenium nanoparticles was elucidated via ultraviolet-visible (UV-Vis) absorption spectroscopy utilizing a PE Lambda 950 spectrophotometer.

### 2.3 Isothermal titration calorimetry experiment

Experiments were conducted at 25 °C on a MicroCal iTC200 isothermal titration calorimeter (GE Healthcare) in PBS buffer (pH 7.4). The titration syringe was loaded with peptide a or scrambled peptide prepared at 500 μM, while the sample chamber contained PD-L1 or HSC70 protein at 2.5 μM. The titration consisted of 20 successive injections of 2 μL each, with an equilibration interval of 120 seconds between injections. The average baseline value was calculated from data points obtained under saturation conditions. Following baseline correction, binding constants were determined using the fitting models provided in MicroCal Origin software.

### 2.4 Cells culture

The Mouse colon carcinoma cell line CT26 was sourced from the Cell Bank of the Chinese Academy of Sciences (Shanghai, China), being maintained in RPMI1640 medium with 10% fetal bovine serum (FBS). Cells are incubated within a controlled environment at 37°C under a humidified atmosphere comprising 5% CO_2_, thereby providing an ideal setting for cellular proliferation and maintenance.

### 2.5 Cellular uptake

CT26 cells were plated in 12-well culture plates and maintained in standard culture conditions until achieving 70-80% confluence. Upon reaching the desired confluency, the existing medium was aspirated and replaced with fresh culture medium supplemented with either FITC-conjugated peptide A or SA. The cells were then incubated for 12 hours under standard cells culture conditions (37°C, 5% CO_2_, humidified environment). After the incubation period, cells were detached using trypsin-EDTA solution and pelleted by centrifugation at 1000 rpm for 5 minutes. To eliminate residual unbound fluorescent molecules, the resulting cell pellets underwent two consecutive washing steps with ice-cold PBS. Following the washing procedure, cells were resuspended in PBS and subjected to flow cytometric analysis to quantify fluorescence intensity and assess the efficiency of cellular uptake.

### 2.6 Validation of PD-L1 degradation

To evaluate PD-L1 expression, CT26 cells were seeded into six-well plates and allowed to attach overnight. Following this initial period, they were subjected to a range of SA concentrations for 48 hours. In order to investigate the underlying degradation pathways, an additional set of CT26 cultures—similarly allowed to adhere overnight—was exposed to SA for 48 h with or without specific inhibitors: the lysosomal inhibitor NH4Cl.

### 2.7 Immunofluorescence colocalization analysis

CT26 cells were seeded on coverslips in 12-well plates (5–6×10^4^ cells/well) and treated with SA (4 μm) for 12 h. After PBS washing, cells were fixed with 4% paraformaldehyde (15 min), permeabilized with 0.2% Triton X-100 (10 min), and blocked with 5% BSA/PBS (1 h, RT). Primary antibodies against PD-L1, HSC70, and LAMP2 (all from Proteintech, 1:200) were applied overnight at 4 °C, followed by Alexa Fluor 594– or 488–conjugated secondary antibodies (1:200, Abcam) for 1 h at RT in the dark. Nuclei were counterstained with DAPI (1:1000, 10 min). Images were captured with a confocal microscope (Olympus FV3000), and colocalization was analyzed using ImageJ with Pearson’s correlation coefficients.

### 2.8 Animal ethics

All mice were obtained from Xi’an Jiaotong university’s laboratory animal center and reared in a controlled, pathogen-free facility, with access to standard feed and a regulated light/dark cycle. Experimental protocols adhered to institutional guidelines and received approval from the same center (ethics approval No.:2022-0059).

### 2.9 Colon cancer subcutaneous tumor model

BALB/c received subcutaneous inoculations of CT26 colorectal cancer cells (1×10^6^ cells per mouse) within the left iliac fossa. Tumor dimensions were diligently monitored using digital calipers, and volumetric calculations were derived utilizing the formula: Volume=length×width^2^/2. Upon reaching approximately 50–100 mm³ in volume, mice were received anti-PD-L1 antibody (mouse monoclonal, clone 10F.9G2™, Bio X Cell) or SA intravenously via lateral tail vein injection at 5 mg/kg on alternate days, while the control group received equal volumes. Following completion of the treatment schedule, tumors were harvested, preserve in formalin, embedded in paraffin, and sectioned for H&E, TUNEL, and various immunohistochemistry assays. The concentrations and group sizes used here were guided by previous findings showing the efficacy of peptide a (28, 29).

### 2.10 Tumor spheroid-immune cell co-culture assay

mCherry-labeled MC38 colorectal cancer cells were generated through lentiviral transduction. Bone marrow-derived dendritic cells (BMDCs) and macrophages (BMDMs) were isolated from BALB/c mice. Tumor spheroids were formed by mixing BMDCs, BMDMs, and mCherry-MC38 cells at a 5:5:2 ratio (500 cells total per well) in 96-well ultra-low attachment plates (Suzhou Santibody Biotechnology). After centrifugation (260×g, 3 min) and 48 h incubation (37°C, 5% CO_2_), spheroids were treated with control medium, anti-PD-L1 antibody or SA (3.19 μg/mL). mCherry fluorescence intensity was monitored at 0, 24, 48, and 72 h using a Celigo Image Cytometer and quantified with ImageJ to assess immune cell-mediated cytotoxicity, with values normalized to baseline.

### 2.11 Flow cytometry analysis

Following euthanasia with 3% isoflurane in oxygen (delivered at 1.5 L/min), tumors were harvested under sterile conditions using sanitized instruments. The specimens were thoroughly rinsed in physiological saline before being finely minced into minute fragments. Subsequently, these tissue pieces underwent digestion in 2 mL of digestive fluid at a temperature of 37°C with orchestration by agitation at a rate of 120 rpm for a duration of one hour. Upon completion of the digestion process, the resultant mixture was filtered through sterile mesh to yield a single-cell suspension, which was then subjected to centrifugation at 700 g for five minutes and reconstituted in Dulbecco’s phosphate-buffered saline.

For the analysis of cytotoxic T lymphocytes (CTLs), cells were stimulated overnight under conditions of 37°C with an atmosphere enriched with 5% CO_2_ utilizing a specialized stimulation cocktail. Post-stimulation, cells were pelleted (700g for 5 min), and subsequently incubated for 40 min with fluorochrome-conjugated antibodies: APC-Cy7 anti-CD45, FITC anti-CD3, and BV605 anti-CD8. After fixation (30 min) and two rounds of lysis/permeabilization, intracellular staining was performed at room temperature for 40 min using BV421 anti-IFN-γ and APC Granzyme B antibodies. Cells were finally resuspended in D-PBS and analyzed by flow cytometry.

Regulatory T cells (Tregs) were detected using a similar protocol—excluding the stimulation step—with surface staining (APC-Cy7 anti-CD45, FITC anti-CD3, BV510 anti-CD4, and APC anti-CD25) and intracellular staining using PE anti-FOXP3. Antibodies were sourced from Invitrogen (Thermo Fisher Scientific).

### 2.12 Immunohistochemical and IF staining

Primary antibodies, such as PD-L1 (Proteintech 66248-1-Ig, 1:5000), CD3 (Servicebio GB15014, 1:10000), CD8 (Servicebio GB15068, 1:10000), and CD4 (Servicebio AB183685, 1:2000), CD25 (GB112325, 1:1000), were diluted in PBS. Samples were incubated overnight at 4°C, and subsequently subjected to washes with PBS. For IF staining, Alexa Fluor 647 or 488 conjugated secondary antibodies at a dilution of 1:200 in PBS supplemented with 1% BSA were added and incubated for 1 h under standard room conditions, shielded from light. For IHC staining, HRP-conjugated secondary antibodies were applied in accordance with the manufacturer’s instructions before proceeding with DAB substrate development. Subsequently, three additional washes with PBS were performed, each lasting five minutes. Nuclei were counterstained with DAPI (Abcam ab104139, 1:10000) for a duration of five minutes under ambient conditions. Finally, the slides were then mounted employing an anti-fade mounting medium and examined under appropriate microscopy systems.

### 2.13 Statistical analysis

For comparisons involving two groups, Student’s *t*-test was applied. Differences among three or more groups were assessed using one-way or two-way ANOVA. Results are reported as mean ± standard deviation (SD). Statistical significance thresholds were defined as *P* < 0.05, *P < 0.01, and ***P < 0.001, with “ns” indicating non-significant differences.

## 3 Result

### 3.1 Schematic illustration of SA design and mechanism of action

**Figure 1:**
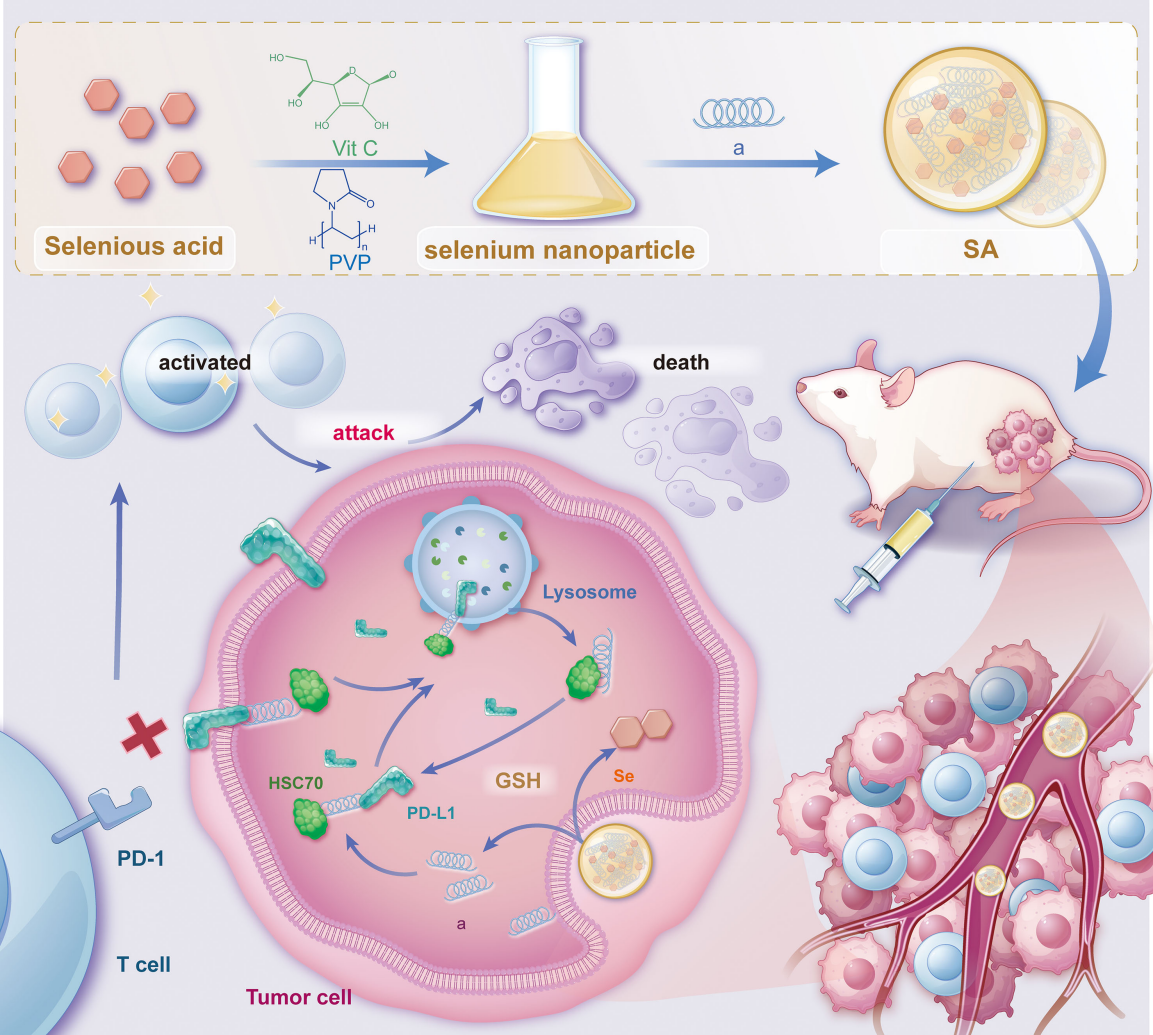
Schematic illustration depicting the synthesis of SA nanoparticles and the intricate mechanism underlying PD-L1 degradation. This schematic outlines the synthesis procedure of SA nanoparticles and their proposed mechanism for promoting PD-L1 degradation via the lysosomal pathway. The process involves the cellular uptake of SA, internalization into endosomal compartments, and subsequent trafficking to lysosomes, where PD-L1 is degraded. Ultimately, this degradation enhances antitumor immune responses by alleviating immune checkpoint inhibition. The schematic was created using Adobe Illustrator.

PD-L1 is commonly overexpressed in various malignancies. After endocytosis, PD-L1 is predominantly sequestered within recycling endosomes and subsequently trafficked back to the plasma membrane, where it continues to inhibit cytotoxic T-cell activity (30). This continuous recycling process has been recognized as a major factor contributing to the resistance observed against PD-L1-blocking immune checkpoint inhibitors (ICIs). As a result, promoting the irreversible intracellular degradation of PD-L1 have gained attention as a promising approach for restoring antitumor immunity (28, 29, 31–33). To achieve this, we have rationally engineered a bifunctional peptide (sequence: CKFERQ-PEG3-nyskptdrqyhf) (**Supplementary Figure S1**) that incorporates (i) a high-affinity motif (KFERQ) for the molecular chaperone HSC70 (34), (ii) a PD-L1-binding sequence (nyskptdrqyhf) (35) and (iii) a PEG3 linker to spatially separate the two motifs and minimize steric interference. Isothermal titration calorimetry (ITC) confirmed that the bifunctional peptide binds to HSC70 with a Kd = 1.28 ± 0.74 μM and to PD-L1 with Kd = 0.41 ± 0.19 μM (**Supplementary Figure S2A**). In contrast, a mutant peptide with scrambled sequences (fhyqrdtpksyn-PEG3-QREFKC) showed no detectable binding to either target (**Supplementary Figure S2B**), confirming the specificity of these interactions. Notably, given the 77% sequence identity and high structural conservation between human and mouse PD-L1 (36), coupled with documented cross-reactive binding between species (37, 38), this peptide is expected to exhibit cross-species activity, supporting the translational relevance of our preclinical murine models. Upon internalization, this peptide concurrently interacts with PD-L1 and HSC70, thereby commandeering the chaperone-mediated autophagy machinery and redirecting cytosolic PD-L1 to lysosomes for degradation. By eliminating the intracellular reservoir of PD-L1, this strategy effectively prevents its re-secretion to the cell surface, thereby attenuating sustained immunosuppressive signaling. However, peptide therapeutics are often hindered by rapid proteolysis, poor serum stability, and limited membrane permeability (39). To address the pharmacokinetic challenges, we utilized the intrinsic biocompatibility and redox characteristics of nano-selenium as a carrier (40, 41). Through selective covalent and electrostatic interactions with lysine, cysteine, and other nucleophilic residues, the peptide self-assembles with nano-Se into a robust, monodisperse nanocomposite, termed SA. This nano-architecture (i) protects the peptide from protease degradation, (ii) enhances cellular uptake, and (iii) leverages the tumor’s oxidative microenvironment for targeted release. Collectively, SA integrates peptide-guided molecular targeting with nano-Se-enabled delivery, establishing a multifunctional platform that accelerates HSC70-dependent lysosomal degradation of PD-L1, disrupts immune-evasion mechanisms, thereby augmenting the effectiveness of cancer immunotherapy (**Figure 1**).

### 3.2 Synthesis and characterization of SA

**Figure 2:**
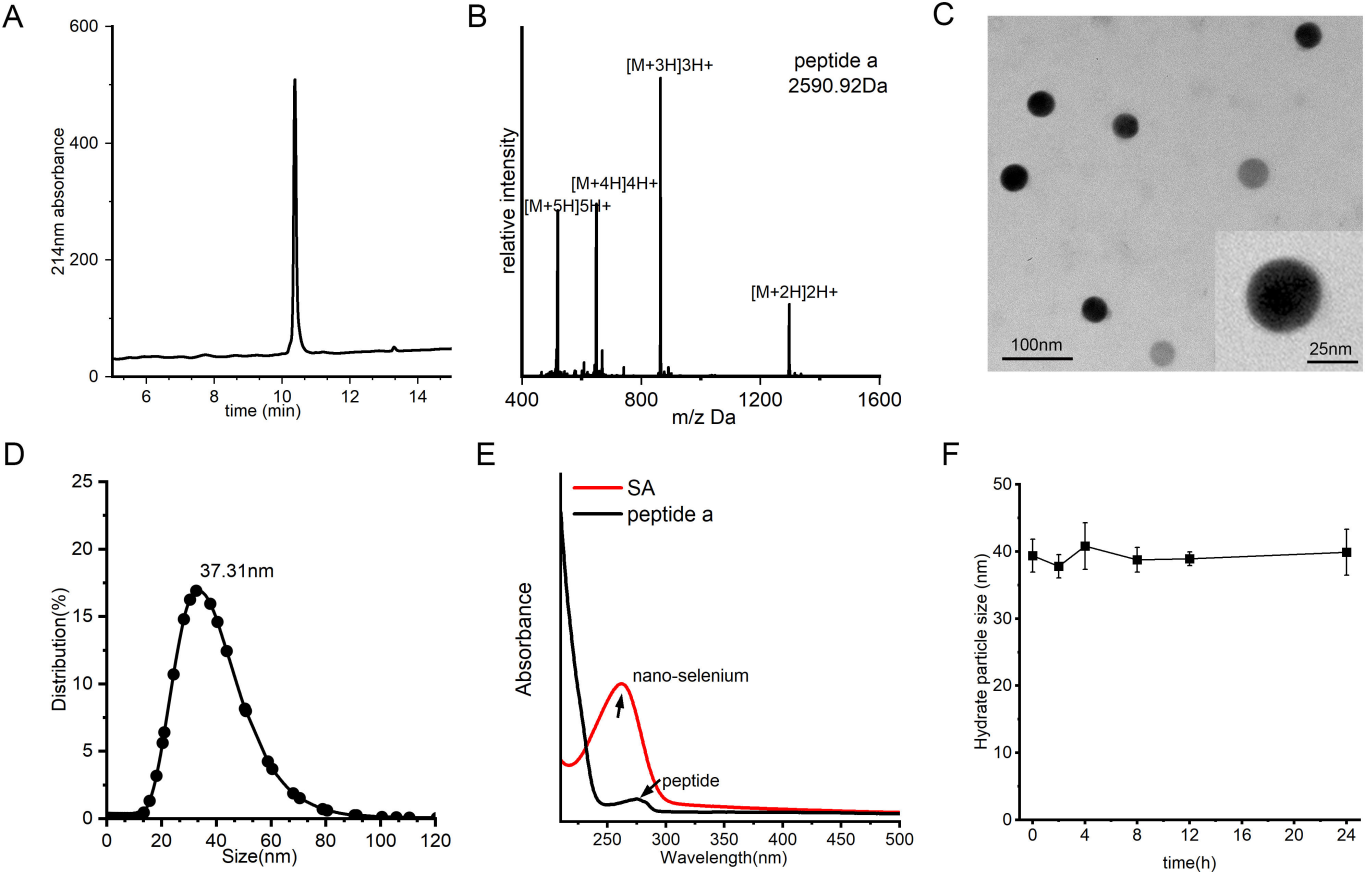
Synthesis and characterization of SA. **(A)** HPLC profile of peptide a, acquired by a C18 reverse-phase column with acetonitrile/water (0.1% TFA) mobile phase under 10-55% acetonitrile linear gradient over 20 minutes for purity assessment. **(B)** Mass spectrometry (MS) spectrum of peptide a, confirming its molecular weight. **(C)** TEM image of SA nanoparticles, revealing their uniform spherical morphology and well-dispersed structure. **(D)** DLS analysis of size distribution of SA. **(E)** Characteristic UV-Vis absorption profile of SA, acquired between 200–800 nm with a 1-cm path length cuvette. **(F)** Evaluation of SA colloidal stability over time in PBS (pH 7.4) containing 20% FBS under physiological conditions (37°C).

To fabricate the SA nanocomposite, we initially synthesized peptide a using a conventional Fmoc solid-phase peptide synthesis (SPPS) protocol as previously reported (42–47). The crude product underwent purification by semi-preparative C18 column and subsequently lyophilized to obtain a white crystalline powder. Analytical HPLC confirmed a high purity of greater than 95%, while ESI-MS validated the molecular identity by displaying the anticipated multicharged ion peaks at m/z 1296.91, 864.64, 649.66, and 520.1, corresponding to [M + 2H]^2+^, [M + 3H]^3+^, [M + 4H]^4+^, and [M + 5H]^5+^, respectively (**Figures 2A, B**). SA was subsequently synthesized through a single-pot reaction in which sodium selenite was reduced by ascorbic acid. Owing to the strong interaction between selenium and nucleophilic residues, peptide a readily self-assembled with nano-Se via selective covalent and electrostatic interactions involving amino and thiol groups, resulting in the formation of a stable nanocomposite, referred to as SA. TEM analysis demonstrated that SA particles were uniformly shaped, predominantly spherical, and monodisperse, with an average diameter of approximately 35 nm, closely aligning with the hydrodynamic size determined by dynamic light scattering (**Figures 2C, D**). The conjugation was further corroborated by ultraviolet-visible spectroscopy, as the characteristic peptide absorption bands disappeared following nano-Se integration, indicating successful binding (**Figure 2E**). Moreover, the SA nanocomplex maintained remarkable stability, exhibiting a consistent hydrodynamic diameter of 35–40 nm in aqueous solution over 24 hours without significant size increase or precipitation (**Figure 2F**). This exceptional colloidal stability under physiological conditions provides a solid foundation for subsequent *in vivo* delivery applications.

### 3.3 SA Could internalize into cells and promote lysosome-dependent PD-L1 degradation *in vitro*

**Figure 3:**
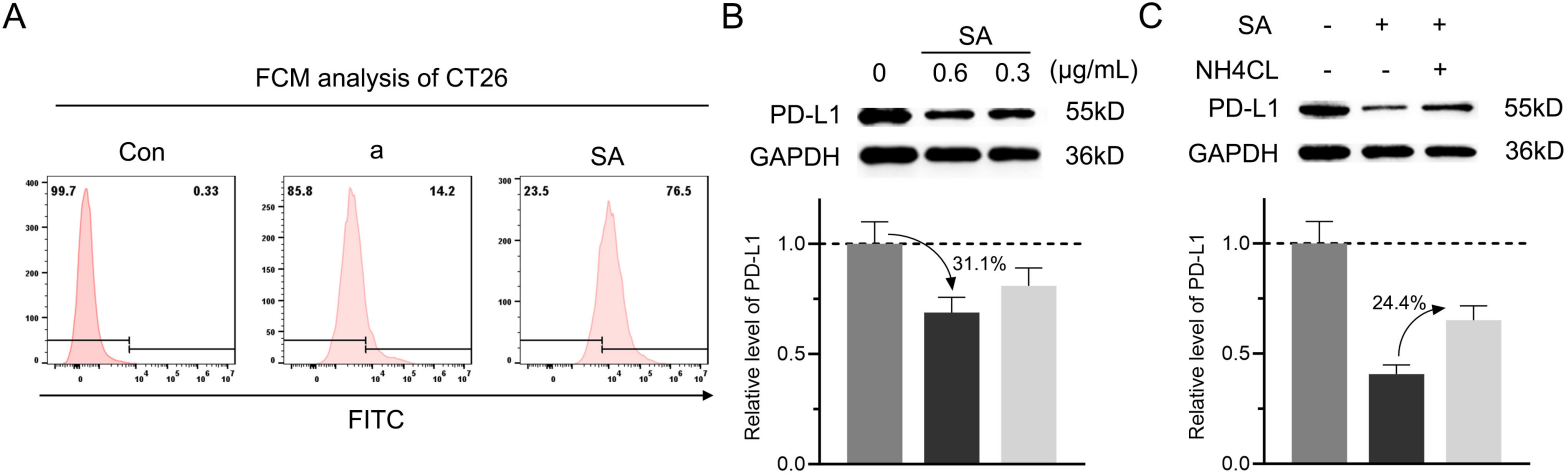
SA could internalize into cells and facilitate PD-L1 degradation through a lysosomal pathway. **(A)** Flow cytometry analysis evaluation of FITC-labeled peptide a or SA uptake in CT26 cells, showing fluorescence intensity distribution. **(B)** Concentration-dependent reduction of PD-L1 in SA-treated CT26 cells (0, 0.6, 0.3 μg/mL), revealed by western blot and quantified through grayscale intensity analysis. **(C)** PD-L1 levels in CT26 cells exposed to SA with or without NH4Cl (6 mM, lysosomal inhibitor), as assessed by western blotting and quantified through grayscale intensity analysis.

Given the poor membrane permeability of peptides, we first examined whether SA could facilitate cellular internalization. Flow cytometry analysis revealed that SA demonstrated significantly enhanced cellular uptake compared to peptide a alone in CT26 colorectal cancer cells, indicating that SA effectively overcomes the membrane penetration barrier faced by the peptide (**Figure 3A**). With successful cellular internalization established, we next evaluated the effect of SA on intracellular PD-L1 protein levels through immunoblotting analysis. Treatment with SA for 24 hours resulted in a dose-dependent reduction of PD-L1, where 0.3 and 0.6 μg/mL resulted in approximately 19.1% and 31.1% decreases respectively, while GAPDH levels remained constant, confirming specific targeting of PD-L1 (**Figure 3B**). To determine whether this degradation occurs through a lysosome-dependent pathway, consistent with extensive evidence suggesting PD-L1 degradation via CMA directed by HSC70 (15, 32), we treated cells with NH4Cl, a lysosome inhibitor that disrupts autophagy function. Co-treatment with NH4Cl significantly attenuated SA-induced PD-L1 degradation by 24.4%, restoring PD-L1 expression levels and confirming the lysosome-dependent nature of this process (**Figure 3C**). To further validate the HSC70-recruited, confocal microscopy revealed that SA treatment significantly enhanced colocalization of PD-L1 with HSC70 and LAMP2-positive lysosomes compare to control group (**Supplementary Figure S3**). These results provide direct visual evidence that SA recruits HSC70 to facilitate PD-L1 trafficking to lysosomes for degradation. Collectively, these findings demonstrate that SA not only solves the membrane permeability issue of the peptide but also efficiently facilitates PD-L1 degradation, mainly mediated by the lysosomal pathway.

### 3.4 SA effectively suppresses tumor growth across tumor models

**Figure 4:**
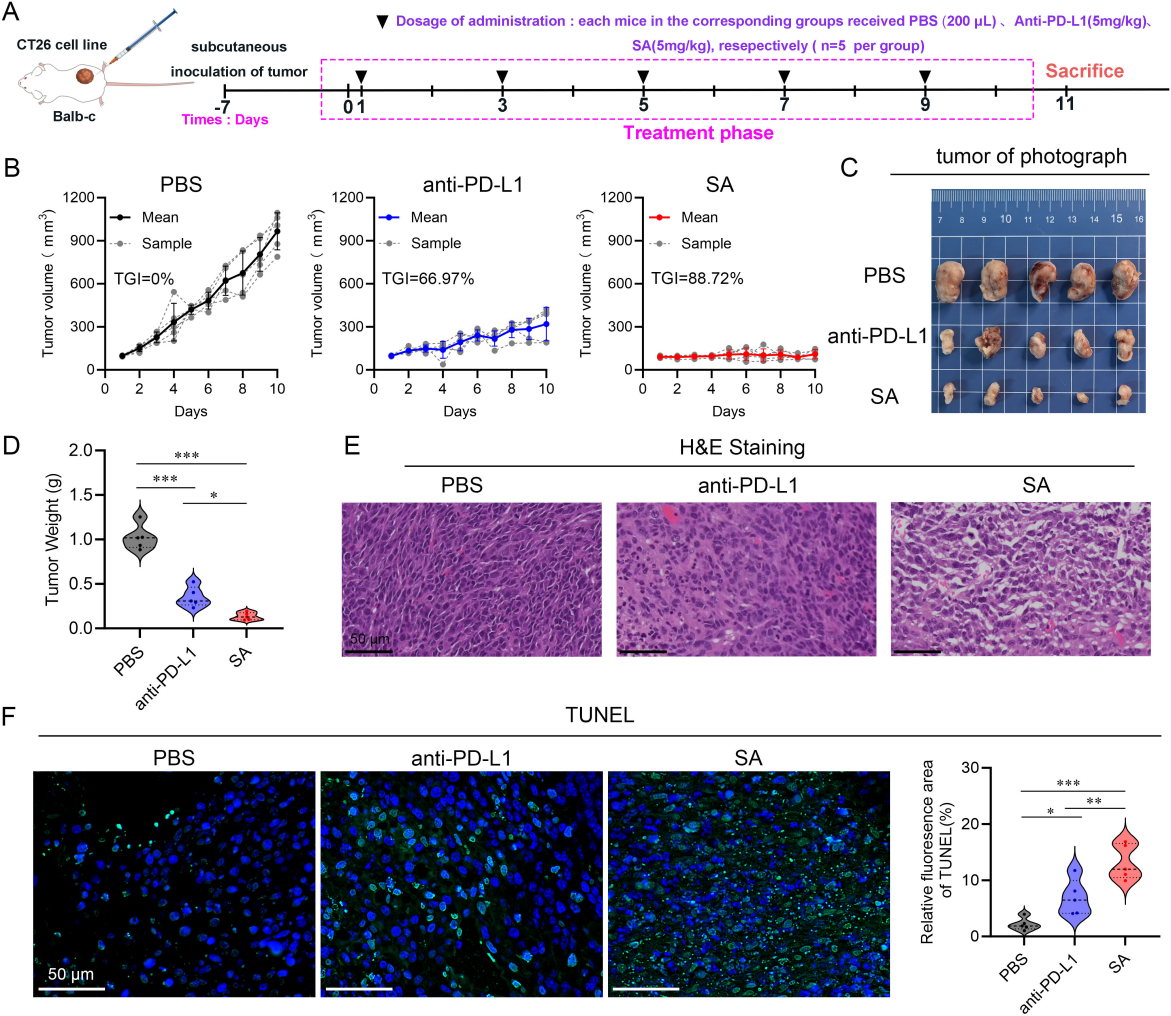
SA suppresses tumor growth in a CT26 subcutaneous colon cancer mouse model. **(A)** A schematic representation of the experimental treatment protocol is depicted. **(B)** Tumor volume trajectories showing changes in tumor volume over the treatment period for each indicated group (n=5, per group). **(C)** Representative photographs of excised tumors from BALB/c mice bearing subcutaneous CT26 tumors following different treatments. **(D)** Quantification of tumor mass at the study endpoint. **(E)** Histopathological analysis by H&E staining of tumor sections derived from PBS, PD-L1, and SA treatment groups. **(F)** TUNEL staining for detection of apoptotic cell in tumor tissue sections from PBS, PD-L1, and SA treatment groups. Left panels display fluorescence microscopy images showing apoptotic cells (green) and cell nuclei (blue, DAPI staining). Right Quantitative assessment of TUNEL-positive cell percentage based on fluorescence area analysis. (scale bar: 50 μm). (*p < 0.05; **p < 0.01; ***p < 0.001).

To comprehensively evaluate the antitumor therapeutic potential of SA, a CT26 colorectal carcinoma syngeneic model was established in BALB/c mice. Upon subcutaneous tumor establishment with volumes ranging from 50–100 mm³, animals were systematically randomized into three experimental cohorts (n=5 per group): a control cohort administered phosphate-buffered saline (PBS), a positive control group receiving anti-PD-L1 monoclonal antibody (5 mg kg^-1^), and experimental group receiving SA treatment (5 mg kg^-1^). All therapeutic interventions were administered intravenously on an alternate-day dosing schedule, with a total of five doses delivered over the study duration (**Figure 4A**). Throughout the 10-day treatment period, continuous tumor volume monitoring revealed distinct therapeutic responses across all treatment groups. While both active treatment modalities demonstrated measurable antitumor activity relative to the PBS control, SA treatment exhibited markedly superior efficacy. Quantitative analysis revealed that SA achieved an impressive tumor growth inhibition (TGI) rate of 88.72%, representing a substantial improvement over the conventional anti-PD-L1 antibody treatment, which yielded a TGI of 66.97% (**Figure 4B**). Macroscopic examination of harvested tumor specimens at study termination provided compelling visual evidence supporting the volumetric measurements. Tumors from SA-treated animals appeared notably smaller and exhibited altered tissue architecture compared to both control and anti-PD-L1 treated groups (**Figure 4C**). Terminal tumor weight analysis further substantiated these observations, with SA-treated tumors demonstrating significantly reduced mass compared to other treatment groups, providing additional quantitative confirmation of the enhanced therapeutic efficacy (**Figure 4D**). Histopathological analysis further corroborated the efficacy results: hematoxylin-and-eosin staining of tumors from the PBS group revealed dense, intact architecture with minimal necrosis, while treatment with the anti-PD-L1 antibody induced moderate structural disruption and scattered apoptotic bodies (**Figure 4E**). In contrast, SA-treated tumors exhibited extensive tissue disorganization, markedly reduced cellularity, and widespread apoptosis confirmed by strong TUNEL positivity (**Figure 4F**). These results demonstrate that SA delivers significantly greater tumor suppression and pro-apoptotic activity than conventional PD-L1 blockade in CT26 models. Moreover, to assess SA’s generalizability beyond the therapeutically resistant CT26 model, we performed spheroid co-culture assays using MC38 cells, an MSI-H model typically responsive to checkpoint blockade (**Supplementary Figures S4A, B**). SA significantly enhanced immune cell-mediated cytotoxicity compared to control and PD-L1 antibody treatments, with pronounced effects at 48h and 72h (p < 0.001) (**Supplementary Figure S4C**). These findings demonstrate that SA maintains superior efficacy across tumor types with distinct immunogenicity, supporting its broad therapeutic applicability.

### 3.5 SA Remodels the tumor immune microenvironment and inhibits tumor progression

**Figure 5:**
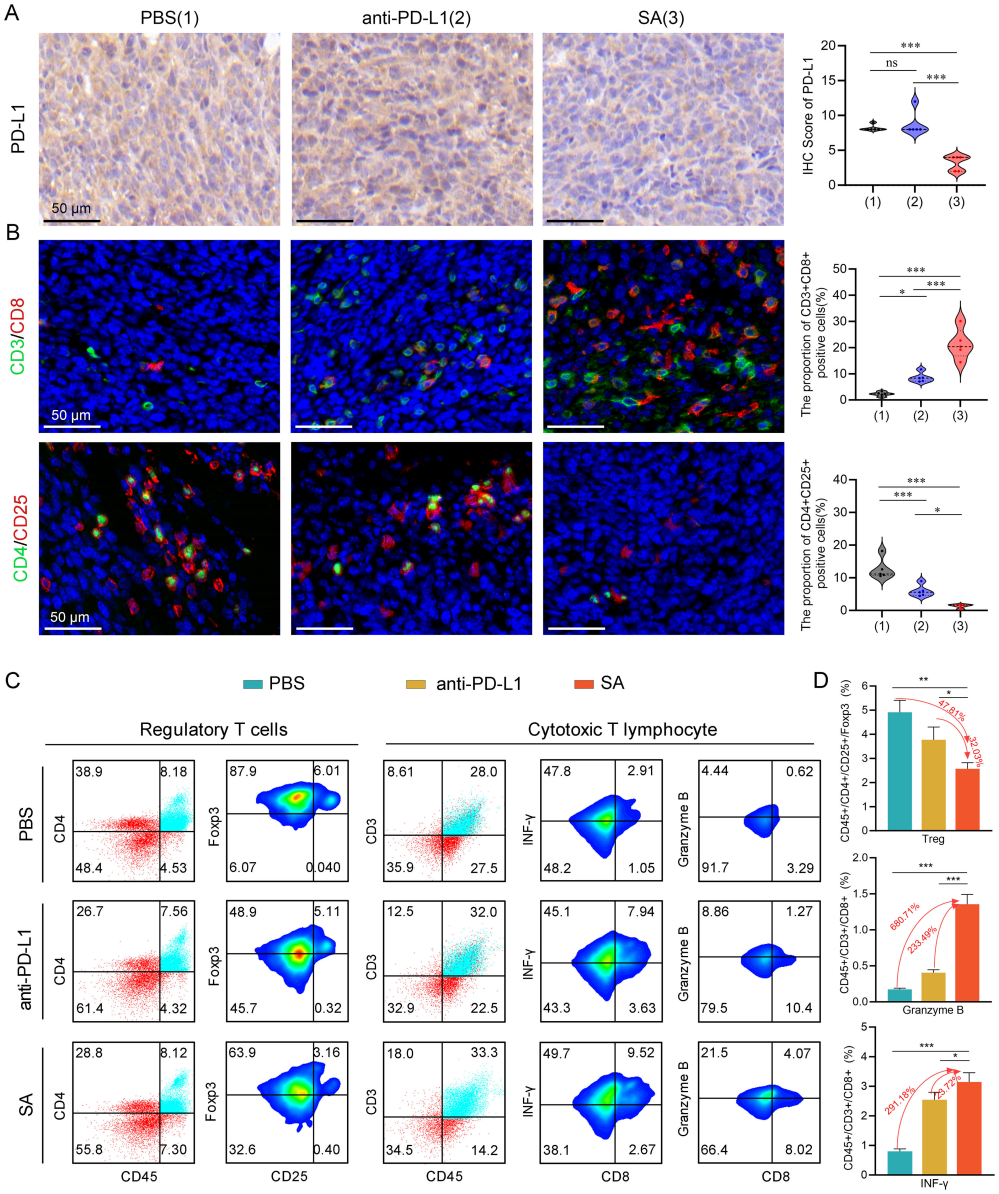
SA remodels the TME and augments antitumor immune responses in CT26 cancer. **(A)** PD-L1 protein expression assessed by immunohistochemical analysis with corresponding semi-quantitative scoring (IHC score). **(B)** Representative immunofluorescence images with quantification analysis of tumor-infiltrating Tregs and CTLs within excised CT26 tumors following different treatments (scale bar: 50 μm). **(C)** flow cytometric evaluation of Tregs and cytotoxic T lymphocytes populations within tumor tissues post-treatment. **(D)** Statistical quantification of immune cell subsets as percentages of total tumor-infiltrating lymphocytes, determined by flow cytometry data. Statistical significance indicated as *P < 0.05; **P < 0.01; ***P < 0.001.

Building upon extensive literature demonstrating that PD-L1 undergoes degradation via the CMA pathway, where HSC70 chaperone proteins facilitate the selective lysosomal targeting for proteolytic degradation (15, 32), we designed our experimental approach to leverage this mechanism for therapeutic intervention. To validate our experimental design, we conducted an immunohistochemical analysis to assess PD-L1 protein level within tissues. Our meticulous evaluation unveiled a striking and statistically significant attenuation in PD-L1 protein levels among tumor tissues from the SA-treated cohort when juxtaposed with both control groups (**Figure 5A**). Specifically, tumors from SA-treated subjects demonstrated markedly diminished PD-L1 immunoreactivity across multiple tumor regions, including both the tumor core and invasive margins, when compared to PBS-treated controls and PD-L1 antibody-treated groups. This pronounced downregulation of PD-L1 expression was consistent with SA’s documented mechanism of action involving enhanced lysosome-mediated proteolytic degradation, confirming the successful targeting of the PD-L1/PD-1 immune checkpoint axis through our therapeutic approach.

To comprehensively examine the impact of SA-mediated PD-L1 reduction on the tumor immune microenvironment, particularly T cell responses, we performed immunofluorescence staining to quantitatively evaluate tumor-infiltrating lymphocyte populations and their spatial distribution within the tumor architecture. Our immunofluorescence analysis revealed profound alterations in the composition and functional state of infiltrating immune cells following SA treatment. Notably, SA treatment induced a pronounced elevation of CD3^+^CD8^+^ cytotoxic T lymphocyte presence throughout the tumor microenvironment. Quantitative analysis revealed a 9.4-fold rise in CD3^+^CD8^+^ T cell numbers in SA-treated tumors relative to PBS controls (*P* < 0.001), with enhanced distribution extending from peritumoral regions into the tumor parenchyma (**Figure 5B**). In parallel with enhanced CTLs infiltration, there was a marked decrease in regulatory T cells (Tregs) within the tumor microenvironment (**Figure 5B**). This coordinated alteration in effector and regulatory T cell populations resulted in a markedly improved CD8^+^/Treg ratio, indicating a fundamental shift from an immunosuppressive to an effector-dominant immune milieu that favors sustained antitumor responses.

To provide quantitative validation of our immunofluorescence findings and enable precise enumeration of immune cell subsets, we performed comprehensive multicolor flow cytometric analysis of tumor-infiltrating lymphocytes isolated from fresh tumor specimens. As expected, flow cytometric analysis confirmed that both PD-L1 antibody blockade and SA administration led to a reduction in the proportion of Tregs in the tumor microenvironment, with SA demonstrating the most pronounced inhibitory effect (**Figures 5C, D**). Specifically, SA treatment reduced the proportion of Tregs by 47.81% compared to PBS controls (p < 0.001), while PD-L1 antibody treatment achieved a 15.78% reduction (p < 0.01). This enhanced depletion of immunosuppressive Tregs by SA treatment suggests a mechanism beyond simple checkpoint blockade, potentially involving direct effects on Treg survival, recruitment, or functional suppression. Beyond quantitative changes in immune cell populations, we next investigated whether SA treatment enhanced the effector performance of tumor-infiltrating CTLs. Flow cytometric analysis revealed that SA therapy markedly elevated the infiltration of CD8^+^ T cells along with their production of key effector proteins critical for tumor cell elimination. SA-treated tumors demonstrated a 6.8-fold increase in CD8^+^ T cells producing Granzyme B, a critical serine protease responsible for perforin-mediated target cell lysis (p< 0.001). Additionally, SA significantly enhanced interferon-gamma (IFN-γ) production, a pleiotropic cytokine that promotes Type 1 immune responses and enhances antigen presentation through MHC class I upregulation. The proportion of IFN-γ^+^CD8^+^ T cells increased by 2.9-fold in SA-treated tumors compared to controls (p < 0.001), indicating robust activation of antitumor effector functions (**Figures 5C, D**). Collectively, these results indicate that SA effectively downregulates PD-L1, promotes CD8^+^ infiltration and activation, suppresses immunosuppressive Tregs, and reshapes the TME toward a pro-effector state, thereby contributing to its potent *in vivo* therapeutic efficacy.

### 3.6 Comprehensive safety assessment of SA treatments in experimental subjects

**Figure 6:**
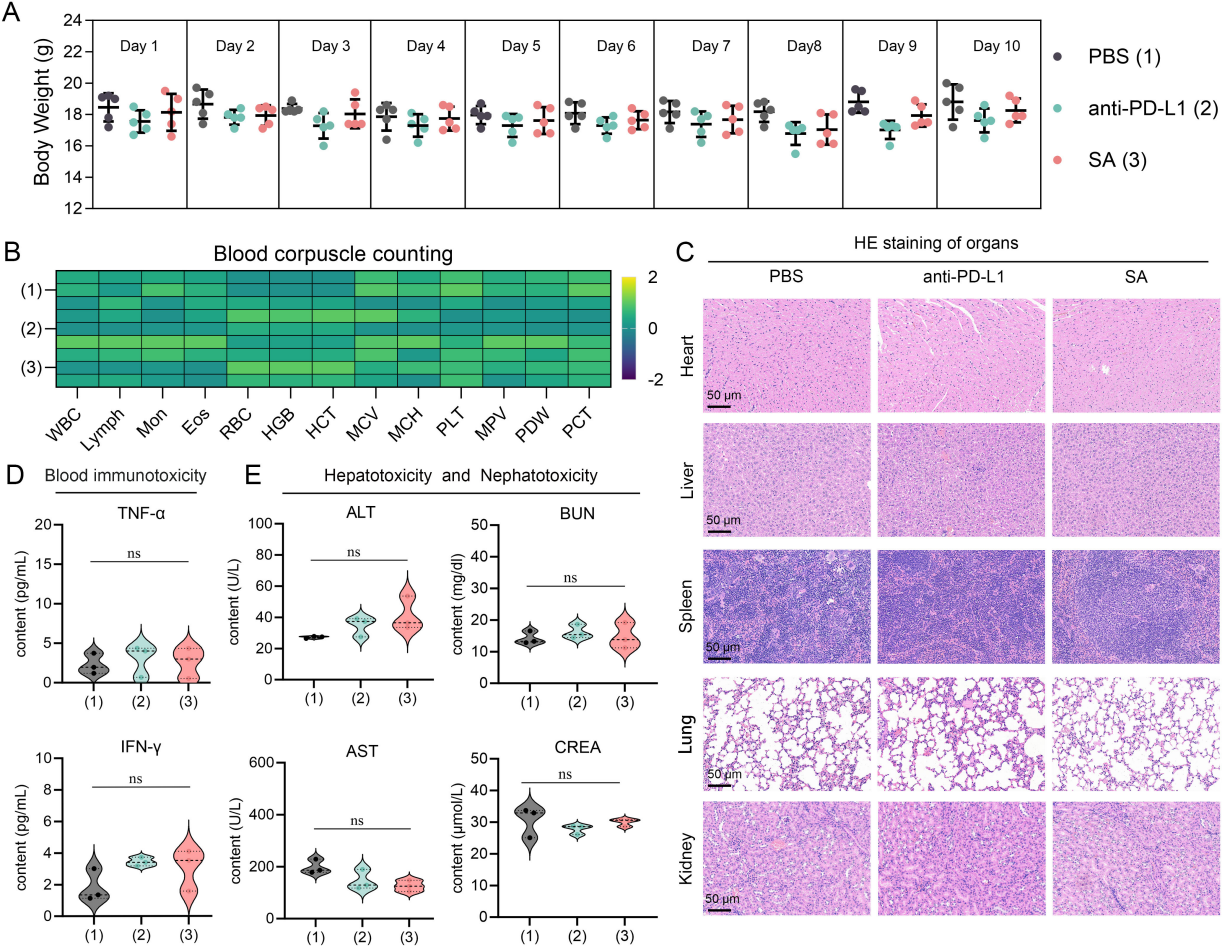
Comprehensive safety assessment of drug treatments in experimental subjects. **(A)** Body weight monitoring over 10-day treatment period showing stable weights across all groups (n=5 per group). PBS (1), anti-PD-L1 (2), and SA (3) treatments. Data presented as mean ± SEM. **(B)** Heatmap of blood corpuscle counting parameters Color scale represents Z-score normalized values. **(C)** Representative H&E staining of major organs (heart, liver, spleen, lung, kidney) from each treatment group. Scale bar: 50 μm. **(D)** Serum inflammatory cytokine levels (TNF-α and IFN-γ) measured by ELISA. **(E)** Hepatotoxicity and nephrotoxicity assessment through serum biomarkers: alanine aminotransferase (ALT), aspartate aminotransferase (AST), blood urea nitrogen (BUN), and creatinine (CREA). All data presented as violin plots with median and quartiles. ns is not significant.

Based on a thorough and meticulous safety evaluation, SA treatments exhibited exceptional tolerability profiles, with no discernible adverse effects. Throughout the entire 10-day treatment period, all experimental groups showcased remarkable physiological stability. Body weight measurements—serving as a primary indicator of overall health status—remained consistently stable across all treatment cohorts. Statistical analyses revealed no significant inter-group differences when compared to PBS control subjects (**Figure 6A**), indicating that neither PD-L1 nor SA treatments elicited systemic stress responses or metabolic disturbances typically manifested as fluctuations in body weight. A comprehensive hematological analysis provided compelling evidence of treatment safety at the systemic level. Complete blood count parameters—including red blood cell count, white blood cell count with differential, platelet count, hemoglobin levels, and hematocrit values—all remained firmly within established physiological ranges across the various treatment groups (**Figure 6B**). This suggests that the treatments did not induce hematotoxicity, bone marrow suppression, or inflammatory responses capable of altering circulating blood cell populations.

Furthermore, detailed microscopic examinations of major organ systems revealed preserved tissue architecture and cellular integrity throughout all treatment groups. Critical organs—including those within the cardiovascular system (heart), hepatic system (liver), immune system (spleen), respiratory system (lungs), and renal system (kidneys)—exhibited no signs of treatment-related pathological alterations (**Figure 6C**). Specifically, no inflammatory cell infiltration, tissue necrosis, fibrosis, or other degenerative changes were observed, indicating that both therapeutic interventions maintain excellent biocompatibility without inducing organ-specific toxicity. Serum biomarker analysis yielded pivotal insights into the immunological safety profile of the treatments. Measurement of key inflammatory cytokines, including tumor necrosis factor-alpha (TNF-α) and interferon-gamma (IFN-γ), revealed levels comparable to those observed in control groups (p > 0.05) (**Figure 6D**). This finding is particularly significant as it demonstrates the absence of systemic immune activation or cytokine storm responses, which are critical safety concerns for immunomodulatory therapies. The maintained cytokine homeostasis suggests that the treatments do not trigger inappropriate inflammatory cascades or systemic immunotoxicity.

Comprehensive evaluation of hepatic and renal function parameters confirmed the preservation of critical organ systems. Hepatocellular integrity markers, including alanine aminotransferase (ALT) and aspartate aminotransferase (AST), showed no statistically significant elevation relative to control values (**Figure 6E**). Similarly, renal function indicators, specifically blood urea nitrogen (BUN) and serum creatinine levels, remained within normal ranges, demonstrating that neither treatment induced hepatotoxicity or nephrotoxicity. These findings are particularly important given that the liver and kidneys are primary sites of drug metabolism and elimination, making them vulnerable to treatment-related toxicity. Collectively, this body of evidence indicates that these therapeutic interventions manifest favorable safety profiles amenable to further preclinical exploration and imminent clinical translation.

## 4 Discussion

Current monoclonal antibody-based ICB therapies aimed at PD-1/PD-L1 grapple with well-documented limitations that significantly restrict their clinical efficacy and broad applicability (48). These constraints include poor tumor tissue penetration, inability to target intracellular immune checkpoints, high immunogenicity, severe immune-related adverse events (irAEs), and the necessity for intravenous administration (49–51). Most critically, conventional PD-L1 antibodies fail to eliminate the intracellular reservoir of PD-L1, which continuously recycles to the plasma membrane and sustains immunosuppressive signaling, contributing to resistance against immune checkpoint blockade therapies. This study addresses these fundamental challenges by developing SA, a rationally designed nano-selenium-peptide platform that promotes comprehensive intracellular degradation of PD-L1 through CMA pathway activation.

The core innovation of SA lies in its bifunctional peptide design that simultaneously engages cytosolic PD-L1 and the molecular chaperone HSC70, enabling targeted degradation of the intracellular PD-L1 reservoir through the CMA pathway. Our mechanistic validation demonstrates dose-dependent PD-L1 reduction (up to 31.1% at 0.6 μg/mL) that is significantly attenuated by lysosomal inhibition (24.4% restoration with NH_4_Cl), confirming the CMA-dependent mechanism. Combined with immunohistochemical evidence of marked PD-L1 downregulation in SA-treated tumors, these findings establish that SA represents a paradigm shift from surface receptor blockade to comprehensive intracellular checkpoint protein elimination.

To contextualize SA’s efficacy across tumor settings with different levels of checkpoint inhibitor responsiveness, we assessed activity in both the therapeutically resistant CT26 (pMMR/MSS) model and the checkpoint-responsive MC38 (MSI-H) model. SA achieved 88.72% TGI in CT26 tumors, substantially outperforming conventional anti-PD-L1 antibody treatment (66.97%), while complementary MC38 spheroid studies confirmed superior efficacy in an MSI-H background. These results suggest that SA retains activity in both resistant and responsive contexts, providing proof-of-concept for cross-tumor efficacy. Future *in vivo* comparative studies in CT26 and MC38 will be important to quantitatively validate efficacy across this responsiveness spectrum and to refine interpretation of SA’s therapeutic potential in relation to tumor immunogenicity.

The integration of nano-selenium as a delivery platform represents a critical component of SA’s therapeutic success, addressing inherent limitations of peptide-based therapeutics including rapid proteolysis, poor serum stability, and limited membrane permeability. Our characterization studies demonstrate that peptide a readily self-assembles with nano-Se through selective covalent and electrostatic interactions involving amino and thiol groups, forming stable, monodisperse nanoparticles with an optimal size of approximately 35 nm. This size range is particularly advantageous for tumor penetration, as it enables efficient extravasation through leaky tumor vasculature while avoiding rapid renal clearance. The nano-Se platform provides multiple therapeutic advantages beyond simple peptide protection. The excellent colloidal stability observed over 24 hours in physiological conditions ensures consistent drug delivery, while the redox-responsive properties of selenium likely facilitate preferential peptide release within the oxidative tumor microenvironment. Additionally, nano-selenium exhibits intrinsic immunomodulatory and antioxidative functions that may synergistically enhance the overall therapeutic response. These properties collectively overcome the pharmacokinetic challenges that have historically limited peptide-based cancer therapeutics.

Beyond direct PD-L1 degradation, SA treatment resulted in comprehensive remodeling of the immunosuppressive tumor immune microenvironment (TIME), fundamentally altering the balance between effector and regulatory immune responses. Flow cytometric analysis revealed that SA treatment achieved a 47.81% reduction in CD4^+^CD25^+^ regulatory T cells compared to PBS controls, significantly outperforming the 15.78% reduction achieved by conventional PD-L1 antibody treatment. This enhanced depletion of immunosuppressive Tregs suggests that SA’s mechanism extends beyond simple checkpoint blockade, potentially involving direct effects on Treg survival, recruitment, or functional capacity. Concurrent with Treg depletion, SA treatment promoted robust activation and infiltration of CTLs throughout the tumor milieu. Immunofluorescence analysis demonstrated a remarkable 9.4-fold increase in CD3^+^CD8^+^ T cell infiltration, with enhanced distribution extending from peritumoral regions into the tumor parenchyma. More importantly, flow cytometric analysis confirmed that these infiltrating CD8^+^ T cells exhibited enhanced functional capacity, as evidenced by a 6.8-fold increase in Granzyme B expression and a 2.9-fold increase in IFN-γ production. This shift towards a pro-inflammatory, effector T cell-dominated microenvironment represents a fundamental therapeutic advantage, as it not only relieves PD-L1-mediated immunosuppression but actively promotes sustained antitumor immune response. Furthermore, the comprehensive safety evaluation conducted in this study provides strong support for the clinical translation potential of SA.

The successful development of SA establishes a foundation for multiple promising research trajectories and translational opportunities. First, the modular design of the nano-selenium platform allows for the incorporation of additional therapeutic peptides or small molecules, potentially enabling simultaneous targeting of multiple immune checkpoints or oncogenic pathways. Second, combination strategies with existing immunotherapies, such as anti-CTLA-4 antibodies or adoptive cell transfer, could further enhance therapeutic efficacy through complementary mechanisms of immune activation. The broader implications of this work extend beyond PD-L1 targeting to establish a new paradigm for immunotherapy development focused on intracellular checkpoint protein degradation. This approach could be adapted to target other immune checkpoint molecules, including TIM-3, LAG-3, and TIGIT, potentially addressing the complex immunosuppressive networks that limit current therapeutic responses. Additionally, the demonstrated ability to overcome key resistance mechanisms suggests that SA-based therapies could be particularly valuable for treating patients who have developed resistance to conventional checkpoint inhibitors.

## 5 Conclusion

In conclusion, we have developed and evaluated SA, an innovative nano-selenium–peptide assembly designed to addresses a critical limitation in current immune checkpoint blockade (ICB) therapy: the intracellular reservoir and recycling of PD-L1. Through activation of the CMA pathway and simultaneous binding to cytosolic PD-L1 and HSC70, SA effectively overcomes major resistance mechanisms associated with deficiencies. The nano-selenium platform ensures essential stability, efficient delivery, and potentially advantageous immunomodulatory properties. Consequently, this results in a potent therapeutic agent that profoundly degrades PD-L1, modifies the TME to enhance immune activation, and demonstrates significantly improved antitumor efficacy compared to conventional PD-L1 antibodies in preclinical studies. This work provides compelling proof-of-concept for an advanced immunotherapy strategy focused on the degradation of intracellular checkpoint proteins. Future research will investigate the applicability of SA across various tumor types, its potential in combination therapies, and further optimization of the nano-platform for clinical translation.

## Data Availability

The original contributions presented in the study are included in the article/**Supplementary Material**. Further inquiries can be directed to the corresponding authors.
