# Supplementary material for: A bifunctional peptide–selenium nanocomposite for lysosomal degradation of PD-L1 and enhanced cancer immunotherapy
Source: Front Immunol. 2025 Oct 29;16:1678911. doi: 10.3389/fimmu.2025.1678911 (PMC12605528; doi:10.3389/fimmu.2025.1678911)
Supplement: Supplementary file 1 [file DataSheet1.doc]

**Supplementary Materials**

**A bifunctional peptide–selenium nanocomposite for lysosomal degradation of PD-L1 and enhanced cancer immunotherapy**

**Yang Wang**^1,2^, **Jun Feng**^1,2^, **Jin Yan** ^1,2*^, **Weiming You** ^1,2*^, **Siqi Yan** ^1,3*^

^1^Department of Hepatology, The Second Affiliated Hospital of Xi’an Jiaotong University, Xi’an, Shaanxi, China, ^2^Department of Tumor and Immunology in Precision Medical Institute, Western China Science and Technology Innovation Port, The Second Affiliated Hospital of Xi’an Jiaotong University, Xi’an, Shaanxi, China, ^3^Institute for Stem Cell and Regenerative Medicine, The Second Affiliated Hospital of Xi’an Jiaotong University, Xi’an, Shaanxi, China

*** Correspondence:**

Jin Yan, yanjin19920602@xjtu.edu.cn

Weiming You, youweiming1014@xjtu.edu.cn

Siqi Yan, yansiqi92@xjtu.edu.cn

**Supplementary Figure**


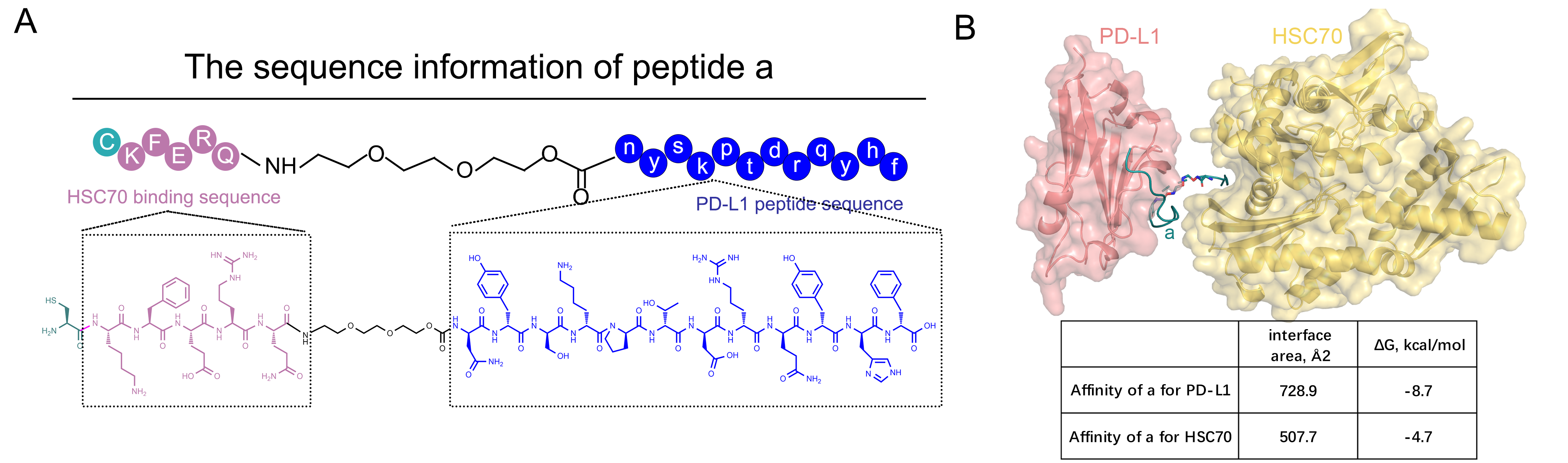


Figure S1. (A) The sequence information of peptide a. (B) The molecular docking of peptide a binding to PD-L1 and HSC70 using the PDBePISA web server.


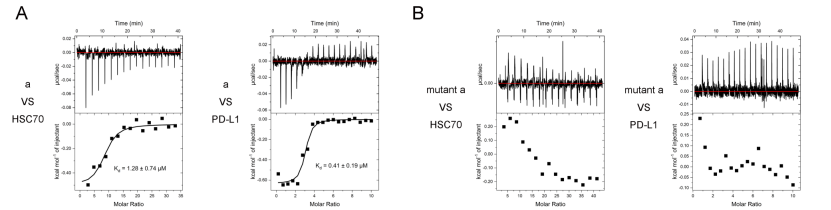


FigureS2 (A) ITC thermogram for a binding to HSC70 and PD-L1. (B) ITC thermogram for scrambled a binding to HSC70 and PD-L1.


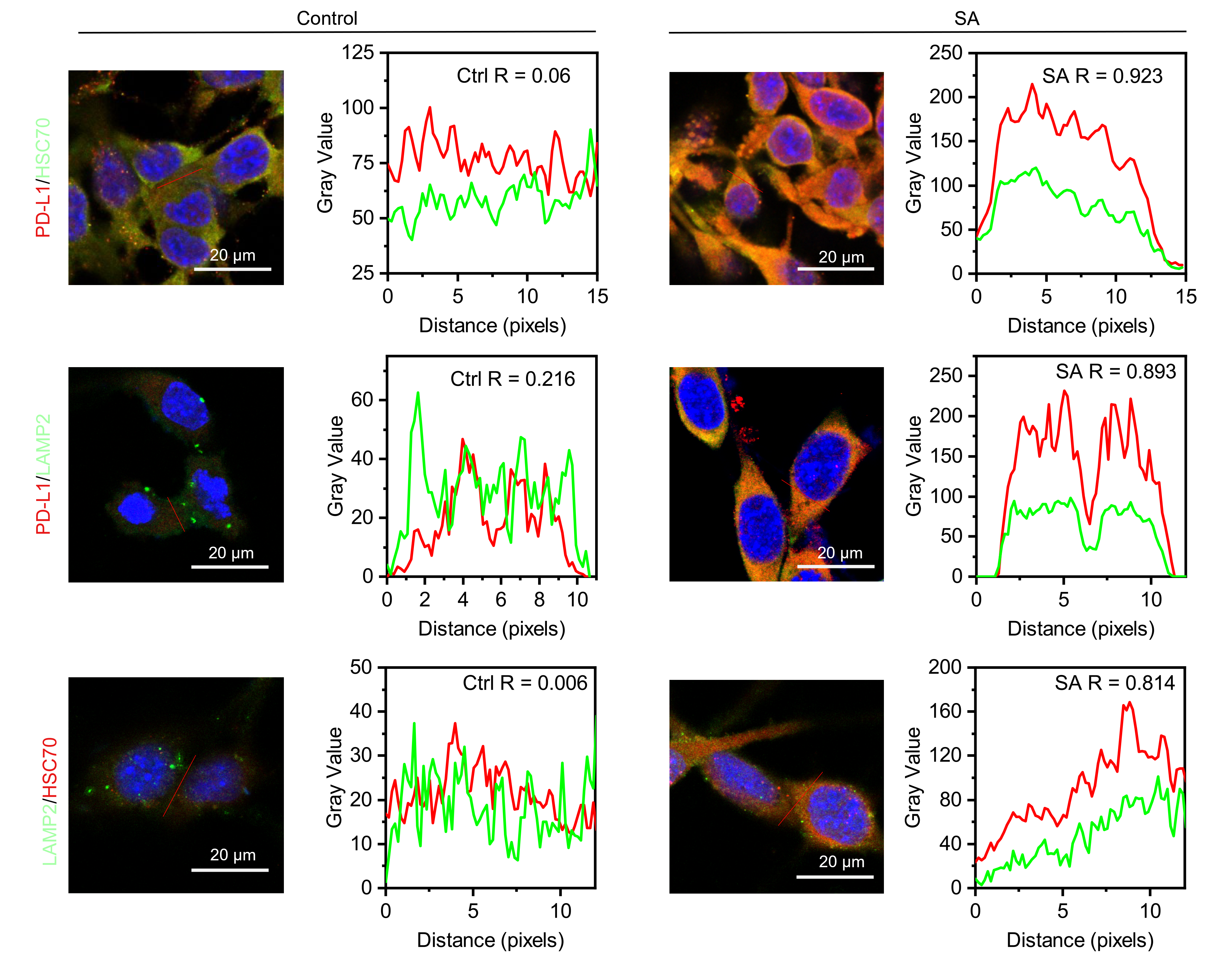


FigureS3 Representative confocal images show the colocalization of PD-L1 with HSC70, PD-L1 with LAMP2, LAMP2 with HSC70 in control and SA-treated cells (12 h). Line-scan analyses along the indicated regions are presented together with Pearson’s correlation coefficients (R values).


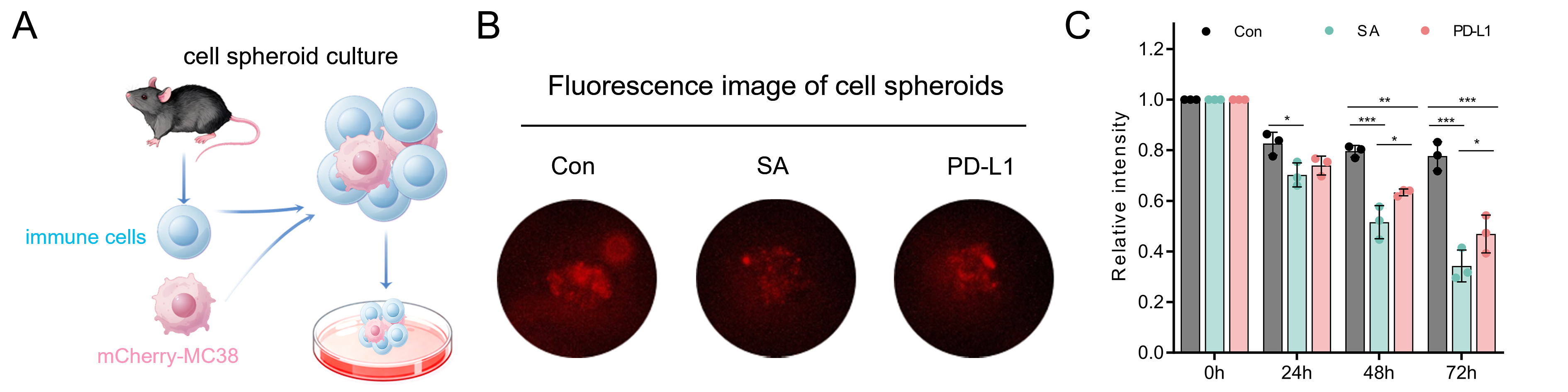


FigureS4 (A) Schematic diagram of experimental design. Immune cells isolated from mouse spleen were co-cultured with mCherry-labeled MCS38 colorectal cancer cell spheroids. (B) Representative fluorescence images of tumor spheroids under different treatment conditions (Control, SA treatment, PD-L1 blockade). Red fluorescence intensity reflects tumor spheroid viability. (C) Quantitative analysis of relative spheroid intensity at different time points (0h, 24h, 48h, 72h) across different treatment groups. Data are shown as mean ± SEM. **P* < 0.5, ***P* < 0.01, ****P* < 0.001.
